# Harmonizing health: a global analysis of pharmaceutical regulatory activities by international regulatory organizations

**DOI:** 10.3389/fmed.2025.1636269

**Published:** 2025-09-23

**Authors:** Agnes Dangy-Caye, Alice Mousset, Adem Kermad, Louise Bouché-Bazerolle, Magda Bujar, Maria-Lucia De Lucia, Neil McAuslane, Rebecca Lumsden

**Affiliations:** ^1^Regulatory Science and Policy Europe, Sanofi, Paris, France; ^2^Centre for Innovation in Regulatory Science, London, United Kingdom; ^3^Regulatory Science and Policy International, Sanofi, Barcelone, Spain; ^4^Regulatory Science and Policy, Sanofi, Reading, United Kingdom

**Keywords:** regulatory organizations, harmonization, reliance, ICH, WHO, ICMRA, IPRP, IMDRF

## Abstract

**Introduction:**

This study demonstrates the role of six international organizations ICH, WHO, PIC/S, IPRP, ICMRA and IMDRF in shaping global health policies and advancing pharmaceutical progress. These six key organizations have been selected based on three criteria: focus on healthcare regulation, international scope, and no geographic restriction on membership. This analysis aimed to map the complementarity of these organizations’ activities.

**Methods:**

For this purpose, a mapping of activities was performed, which identified 10 domains: clinical, convergence and reliance, digital, generics and biosimilars, innovative therapies, medical devices, non-clinical, pharmacovigilance, public health, and quality. Five main types of outputs were also identified: collaborative work, guidance, information, standards and norms, and training.

**Results:**

Key takeaways show that the most active domains among international regulatory organizations are quality, public health, convergence and reliance, and pharmacovigilance. But emerging priorities, such as digital health and innovative therapies, are also captured, demonstrating the regulatory framework is constantly evolving. A focus on one of the domains has been made, convergence and reliance, to demonstrate the impact to be part in one of these international organizations: a detailed analysis showcases the advantages of ICH membership, especially its positive impact on reducing submission lag times for new active substances in member countries. Collaboration between international organizations strengthens global regulatory systems. Our study evaluated the interaction between regional and international memberships. Participation in regional organizations correlated with membership in international organizations, suggesting these memberships facilitate involvement in global regulatory framework activities. Global harmonization of technical standards across regulatory frameworks is extremely challenging. Therefore, additionally a comparison was made between ICH members and non-members to observe any influence of ICH on broader multinational engagement. ICH member countries were found to be more active participants in the international regulatory organizations compared to non-member countries.

**Discussion:**

This research highlights the critical role of international regulatory organizations in harmonizing global regulatory frameworks and fostering pharmaceutical innovation. Their collaborative efforts and synergies contribute to a robust and cohesive regulatory landscape, ultimately benefiting patients worldwide. By promoting cooperation and knowledge sharing, these organizations ensure the safety, efficacy, and quality of medicines and healthcare products on a global scale.

## 1 Introduction

Today, innovation is global, requiring strong and aligned regulatory frameworks to help make both research and development activities and manufacturers’ work easier in the development and supply of medicines. International organizations like the International Council for Harmonization (ICH) plays a key role in harmonizing standards, ensuring efficiencies across regions. This global technical harmonization is challenging and takes a long time, so regulatory strengthening activities also encourage convergence of standards and processes as a move towards full technical harmonization (1, 2). Globally Industry seeks to streamline processes, reducing duplication while maintaining high safety standards. Strengthening global regulatory collaboration is essential to supporting innovation and patient access worldwide.

Rapid access to medicines is a major global challenge. An effective solution is participation in international organizations that harmonize pharmaceutical requirements. To join these organizations, countries must adopt common standards of quality, safety, and efficacy for health products. This approach promotes global standardization, thus facilitating access to medicines for all.

The accessibility of medicines then becomes a crucial national issue for regulatory authorities. They must maintain a delicate balance: on one hand, being demanding on the standards of quality, safety, and efficacy of health products in their territory, and on the other hand, remaining attractive to manufacturers. Indeed, the latter may be discouraged if each marketing authorization application requires different specifications depending on the country. International harmonization helps resolve this dilemma by creating a common framework that benefits both authorities and the pharmaceutical industry.

In this research paper, an investigation of the activities of six multinational regulatory organizations dedicated to medical and healthcare products was made. These organizations were selected based on specific criteria, including their focus on healthcare product regulation, international scope, and inclusiveness across geographic regions. Examining the roles and activities of these organizations, the aims were to clarify their importance in shaping regulatory frameworks, promoting global health, and advancing the collective mission of the pharmaceutical industry.

This article primarily delves into the analysis of activities and outputs of these various international drug regulatory organizations in their pivotal role through which they orchestrate the international regulation of medicines. In addition, it aims to assess whether their efforts are complementary or running in parallel. Alongside, the levels of engagement of different regions worldwide in pharmaceutical regulations are analyzed. To better understand whether greater international engagement might be driving convergence with global standards, and furthering the efficiency and harmonization of regulatory practices, reliance pathway utilization and submission lag trends were analyzed. This comprehensive examination aims to have a general vision of international regulation organizations and their impact on global health and pharmaceutical advancement and to propose some actions to increase convergence and harmonization among regulators.

## 2 Methods

### 2.1 Organizations

The research for this study focused on the activities of six multinational regulatory organizations between January 2018 to June 2024. Regulatory activity was collected to August 2023 and then reviewed and updated to June 2024. All data was drawn from organization websites (see below) – last accessed March 2025.

These organizations were selected based on the following criteria:

- A focus on medicines, medicinal products, or medical devices
- An international scope
- Acceptance of countries worldwide without geographic restrictions

The organizations that were selected are:

- International Council for Harmonization of Technical Requirements for Pharmaceuticals for Human Use or ICH (3): All collected activities come from the official ICH website*: ICH Official web site: ICH*
- World Health Organization or WHO (4): All collected activities come from the official WHO website: *World Health Organization (WHO)*
- Pharmaceutical Inspection Convention (PIC) and Pharmaceutical Inspection Cooperation Scheme (PICS) or PIC/S (5): All collected activities come from the official PIC/S website: *PIC/S*
- International Pharmaceutical Regulators Program or IPRP (6): All collected activities come from the official IPRP website: *IPRP – International Pharmaceutical Regulators Programme*
- International Coalition of Medicines Regulatory Authorities or ICMRA (7): All the activities come from the official ICMRA website: *International Coalition of Medicines Regulatory Authorities (ICMRA)|International Coalition of Medicines Regulatory Authorities (ICMRA)*
- International Medical Device Regulators Forum or IMDRF (8): All the activities come from the official IMDRF website*: International Medical Device Regulators Forum (IMDRF)|International Medical Device Regulators Forum*

### 2.2 Activity mapping by topic domains

To comprehensively understand the scope of activities undertaken by these various regulatory organizations, a detailed analysis of their documented outputs from January 2018 to June 2024 was undertaken. This analysis aimed to map out the various subjects and areas of focus these organizations have been actively engaged in. This approach allowed insights into the breadth and depth of their regulatory activities, providing a clear picture of the issues and challenges they address within the pharmaceutical and medical sectors. By examining their publications over this period, trends, priorities, and developments in the regulatory landscape could be discerned, thus facilitating a deeper understanding of their roles and contributions to global health and safety. The activities were grouped into the following categories:

- Clinical: Activities related to the efficacy of medications, clinical studies, and Real-World Data/Real-World Evidence.
- Convergence and reliance: Activities aligned with the definitions of Convergence and Reliance, as defined in the WHO’s Good Regulatory Practices.
- Digital: Activities related to the objective of digitalization of the pharmaceutical regulatory environment.
- Generics and biosimilars: Activities concerning the regulation of generics and biosimilars.
- Innovative therapies: Activities related to the regulation of innovative treatments, such as nanodrugs, gene therapies, and cell therapies or any new scientific technologies.
- Medical devices: Activities related to medical devices regulation.
- Non-clinical: Activities associated with the safety of medications, such as toxicological studies.
- Pharmacovigilance: Activities related to case reporting and pharmacovigilance.
- Public health: Activities related to public health, such as the fight against pandemics, activities linked to drug shortages or the fight against antimicrobial resistance.
- Quality: Activities pertaining to quality assurance, including Chemistry Manufacturing and Control (CMC), Good Manufacturing Processes (GMP), inspections, norms, and standards.

To streamline data collection, calculations, and visualizations, each project was assigned to a single primary domain representing its main focus by two authors. The classification was then reviewed and validated by two different authors. A key limitation is that some projects could map to more than one category, in these cases a primary grouping was proposed and validated.

### 2.3 Actions mapping by output types

The tasks undertaken by international regulatory authorities cover a broad spectrum of topics, each leading to various outputs. To effectively categorize these outputs, the following grouping was proposed:

- *Collaborative work*: All outputs that are going under this category are actions that foster collaboration among regulatory authorities, such as the establishment of working groups and discussion forums.
- *Guidance*: All outputs that are going under this category aim to develop regulatory frameworks, including the creation or update of regulations, guidelines, guides, and evaluation procedures.
- *Information*: All outputs that are going under this category aim to facilitate the sharing of information within the regulatory community and with healthcare professionals or patients. This may include publications, conferences, and other dissemination efforts.
- *Standards and norms*: All outputs that are going under this category aim at harmonizing and standardizing practices, including work on terminology, formats, and nomenclature.
- *Training*: All outputs that are going under this category are activities which focus on providing training to regulatory and inspection authorities to enhance their skills and knowledge.

To streamline the analysis, a single primary output was assigned to each project by two authors. The classification was reviewed and validated by two different authors. A key limitation is that some projects could map to more than one category, in these cases a primary grouping was proposed and validated.

### 2.4 Geographical analysis

The analysis covers both the representation of countries in the composition of international regulatory organizations and their projects. To analyze the membership of these organizations, the list of countries recognized by WHO, along with the geographical divisions proposed by WHO, were utilized. In addition, the following two jurisdictions, not recognized by WHO but acknowledged by some organizations, were included: Hong Kong and Chinese Taipei.

Some countries are represented by regional organizations in a specific geographical region, which are referred to in this study as “regional harmonization initiatives” (RHI) and will be part of the geographical analysis of the activities of international organizations. As a first step, the number of memberships in regional organizations were analyzed for each region of the world, examining the impact of regional harmonization initiatives.

In a second step, an analysis was carried out and reviewed to assess the influence of participation in one international organization on involvement in other similar organizations, to determine whether member countries of one international organization are also represented in a larger number of other international organizations. For this purpose, the membership of ICH and non-ICH countries in other multinational organizations was analyzed and a Mann–Whitney U test was performed to determine the *p*-value.

### 2.5 Reliance and submission lag

To better understand whether greater international engagement might be driving convergence with global standards, and furthering the efficiency and harmonization of regulatory practices, reliance pathway utilization and submission lag trends were analyzed.

For this, data were sourced from the Growth and Emerging Markets Metrics (GEMM) Programme database maintained by the Centre for Innovation in Regulatory Science (CIRS). The GEMM Programme is an annual benchmarking study collecting data from 14 multinational pharmaceutical companies that seek to better understand the processes and timelines for the registration of medicines in key growth/emerging markets. The database consists of applications to market New Active Substances (NASs) and Major Line Extensions (MLEs) in these markets, in addition to certain characteristics of these applications, the general submissions processes, country-specific processes, and the timelines for from submission to registration. For this study, NAS applications submitted to seven Asian markets (China, India, Indonesia, Malaysia, Singapore, South Korea, and Chinese Taipei) and three additional key growth/emerging markets covered by the Programme (Brazil, Egypt, and Saudi Arabia) were analyzed.

The first part of this analysis focused on the proportion of NAS applications submitted to each growth/emerging market between 2021 and 22 by the assessment route used: verification, abridged, full, other, or unknown. Particular attention was given to the reliance pathways (verification or abridged assessment), and markets with proportionally higher usage of these routes were evaluated to determine whether this correlated with greater engagement in such international organizations as ICH or WHO.

#### 2.5.1 New active substance

A substance not previously authorized as a medicinal product within the concerned jurisdiction.

#### 2.5.2 Full review

A regulatory assessment where a complete dossier is submitted to regulatory authorities to support a full review of quality, safety, and efficacy.

#### 2.5.3 Abridged review

A regulatory assessment that relies on data from previous assessments of a medicine by trusted authorities. It minimizes re-evaluation by focusing on ensuring specific local requirements are met.

#### 2.5.4 Verification review

A more streamlined assessment where the regulator confirms that the product meets established criteria based on prior approvals by reference agencies.

The second part of this analysis examined submission lag, defined as the time between first market approval and submission to the growth/emerging market. A three-year moving median (2004–2023) was used to assess the trend in submission lag in each of the countries, comparing periods before and after the initiation of each jurisdiction’s involvement with ICH (either as a member or observer). This part of the analysis aimed to evaluate whether engagement with international organizations focused on international standards harmonization (i.e., ICH) was correlated with shorter submission lag timelines.

## 3 Results

### 3.1 Activities of international organization

#### 3.1.1 World Health Organization

The World Health Organization (WHO), established on April 7, 1948, is the world’s oldest international health organization (9). Between 2018 and 2024, WHO was involved in 167 projects relating to medicine regulation, all other WHO activities were excluded from this analysis. Table 1 lists WHO projects and their classification by topic domains based on their subject and type, in accordance with the methodology previously outlined.

**Table 1:** WHO topic domains and outputs by project.

Topic domain	Output	Number of projects	Summary of project types	% of WHO activities by topic
Clinical	Guidance	1	Assessment of stability in international collaborative studies	1.2%
Clinical	Standards and Norms	1	Joint Statement on transparency and data integrity ICMRA and WHO	
Convergence and reliance	Collaborative Work	9	Guidelines on regulatory preparedness of pandemic & emergency use, Global and Regional Regulatory Harmonization Initiatives, R&D Blueprint, List of regulatory agencies (WLA) & Access Tools (ACT, C-TAP, GBT.)	16.8%
Convergence and reliance	Guidance	16	Good practices (reliance, collaborative registration procedures, storage and distribution, Regulatory Preparedness and Readiness, quality management systems), Operational guidance on WLA, Guidance on COVID-19 (national deployment and vaccination plan, EUL-FP for *in vitro* diagnostics), Guidance to manufacture non-sterile pharmaceutical products, Global competency framework for regulators of medicines (GCF)	
Convergence and reliance	Information	2	WHO questionnaire analysis - Conferences to discuss collaborative approaches, international consensus, harmonization/convergence on regulation	
Convergence and reliance	Standard and Norms	1	International Nonproprietary Names (INN)	
Digital	Guidance	1	Guideline on data integrity	1.2%
Digital	Standard and Norms	1	Electronic Certificate of Pharmaceutical Product (eCPP)	
Generics and biosimilars	Guidance	4	Guidelines on evaluation of biosimilars (Bioequivalence Studies for Reproductive Health, equilibrium solubility experiments), WHO “Biowaiver List”	3.0%
Generics and biosimilars	Information	1	WHO survey on the evolving regulatory landscape for similar biotherapeutic products	
Innovative therapies	Collaborative Work	1	Regulatory convergence on cell and gene therapy products (CGT)	3.0%
Innovative therapies	Guidance	2	Developing a regulatory framework for human cells and tissues and for ATMPs - Standardization of CGT products	
Innovative therapies	Standard and Norms	2	WHO white paper on regulatory convergence for CGTPs - Update on the standardization of CGT products	
Medical devices	Collaborative Work	1	Collaborative procedure between WHO and national regulatory authorities in the assessment and accelerated national registration of WHO-prequalified *in vitro* diagnostics	10.8%
Medical devices	Guidance	16	WHO Global Model Regulatory Framework for medical devices, WHO/UNPF prequalification program guidance for multiple devices, Eligibility criteria & assessment, Guidance for surveillance of medical devices, Technical Guidance Series on IVD	
Medical devices	Standards and Norms	1	Technical Specifications Series IVD	
Non-clinical	Guidance	1	Review of animal testing requirements in WHO guidelines and recommendations for biological products to implement the 3Rs principles	0.6%
Pharmacovigilance	Collaborative Work	5	Vaccine safety communication, Collaborative Centers (WHO CC), Real-time Monitoring VIgiLyse, Program for International Drug Monitoring, Safety alerts	13.8%
Pharmacovigilance	Guidance	8	Pharmacovigilance Strategies, safety monitoring & surveillance, WHO vaccine reaction rates information sheets, Immunization stress-related response and during pregnancy, Guidance for COVID-19 clinical case management (thrombosis with thrombocytopenia syndrome - TTS, molnupiravir), Landscape analysis in LMIC	
Pharmacovigilance	Information	6	Vaccine Safety Net, Recommendation from Advisory Committees (on Vaccine Safety - GACVS, on Safety of Medicinal Products - ACSoMP, on Vaccine Safety - GACVS), WHO tools (hemovigilance system, Advanced Analytics &Visualization)	
Pharmacovigilance	Standard and Norms	3	Investigation and causality assessment of adverse events, Innovative decision-making electronic tools, Active surveillance (CIOMS), Global COVID-19 Clinical Platform, WHO Guidelines to assure the quality, safety and efficacy of live attenuated rotavirus vaccines (oral)	
Pharmacovigilance	Training	1	Training resources	
Public health	Collaborative Work	6	Pediatric Regulatory Network (PRN), SF Medical Product, Medical Product Alerts, Vaccines Advisory Committee, ICMRA/WHO Global perspectives on COVID-19 vaccines strain update	26.3%
Public health	Guidance	26	Guidelines to assure the quality, safety and efficacy of various vaccines (rotavirus, poliomyelitis, yellow fever, typhoid, enterovirus 71, respiratory syncytial virus, recombinant hepatitis E, influenza viruses), and messenger RNA & plasmid DNA vaccines for the prevention of infectious diseases, Guidelines on the nonclinical and clinical evaluation of mAb and related products intended for the prevention or treatment of infectious diseases, COVID-19 and of respiratory syncytial virus, WHO guidance and Action framework for an universal and adequate access of quality-assured blood, particularly during pandemic Global Framework to Combat Antimicrobial Resistance, falsified medicines, Selection and Use of Essential Medicines and Medical Product Alerts	
Public health	Information	2	Stakeholders in COVID-19 vaccines safety surveillance - Statement for healthcare professionals: How COVID-19 vaccines are regulated for safety and effectiveness	
Public health	Standard and Norms	9	International collaborative study: Assessment of commutability, Global assess (COVID-19), ethical considerations, International Classification of Diseases (ICD 11), Calibration of working reagents by WHO essential regulatory laboratories for seasonal antigens, Guidelines for the production and quality control of monoclonal and Cell banks (Vero), WHO-ICMRA joint statement to improve global regulatory alignment on COVID-19 medicines and vaccines - Report on the review of regulatory flexibilities/agilities as implemented by National Regulatory Authorities during Covid-19 pandemic - Deep dive report on the review of provisions and procedures for emergency authorization of medical products for COVID-19 among ICMRA members	
Public health	Training	1	Educational modules on clinical use of blood – first tranche	
Quality	Collaborative Work	4	Strategy, Policy and Partnership of LPA Unit, Technology Transfer Facilitation, Update on activities related to COVID-19, Laboratory Networks and Services	23.4%
Quality	Guidance	25	Good practices on multiple subjects (Chromatography, water for pharmaceutical use, pharmacopoeia monographs, manufacture of compounded preparations & herbal medicines, desk assessment of compliance with GMP - GLP - GCP, on Radiopharmaceuticals with IAEA & International Atomic Energy Agency, for blood establishments, validation, medicinal gases, for investigational products, for sterile pharmaceutical products, for research and development facilities, technology transfer, reference materials for use as secondary standards in antibody testing, Stability testing of APIs and finished products, requesting analysis of medicines samples.)	
Quality	Information	1	Situational Analysis and Readiness of LPA Unit	
Quality	Standard and Norms	5	Model certificate of analysis, International Pharmacopoeia, Deletion of the innocuity/abnormal toxicity test for biological products, International Reference Materials, Identification of Medicinal Product Working Group (IDMP)	
Quality	Training	4	Capacity Building and Technical Assistance for WHO Prequalification and for Laboratories (LNS), Training Workshop on Key Enabling Factors for Successful Local Production and Supply of Quality-Assured Medicines in Africa,	

Of the 167 WHO-mapped projects, 39 (23.4%) are related to quality, 44 (26.3%) to public health, 28 (16.8%) to Convergence and Reliance, and 23 (13.8%) to Pharmacovigilance. Additionally, WHO offers activities on digital, innovative therapies, and Generics & Biosimilars.

The WHO outputs primarily take the form of Guidance (59.9%), Collaborative work (15.6%) and Standards and Norms (13.8%). WHO also provides information and training as outputs, but in smaller quantities.

#### 3.1.2 International Council for Harmonization

The ICH projects examined were categorized in accordance with the methodology and illustrated in Table 2.

**Table 2:** ICH topic domains and outputs by project.

Topic	Output	Number of projects	Summary of project types	% of ICH activities by topic
Clinical	Guidance	10	E11A EWG - E20 EWG - E21 EWG - E22 IWG - E6(R3) EWG - E8(R1) - E9 (R1) - Incorporating Patient Experience to Better Inform Drug Development and Regulatory Decision Making (PFDD) - Pursuing Opportunities for Harmonization in Using Real-World Data to Generate Real-World Evidence, with a focus on Effectiveness of Medicines - Strategic approach to harmonization of Technical Scientific Requirements for Pharmacoepidemiological Studies	18.0%
Clinical	Training	1	E14/S7B	
Convergence and reliance	Guidance	2	Cell and Gene Therapies Discussion Group (CGTDG) - Memorandum of Understanding (MoU) between PIC/S and ICH	3.3%
Digital	Guidance	4	M11 - M15 - M2 EWG - M8	6.6%
Generics and biosimilars	Guidance	5	Harmonize standards for generic drugs - M13A EWG - M13B EWG - M13C EWG - M9	8.2%
Innovative therapies	Guidance	1	Advancing Biopharmaceutical Quality Standards	1.6%
Non-clinical	Guidance	9	M12 - M7 Subgroup - M7(R2) - S11 - S12 - S13 EWG - S1B(R1) - S1B(R1) IWG - S5(R3) + S5(R4)	14.8%
Pharmacovigilance	Guidance	4	E19 - E2B(R3) EWG/IWG - E2D (R1) EWG - M14 EWG	8.2%
Pharmacovigilance	Standards and Norms	1	M1 PTC WG	
Public health	Standards and Norms	1	International Classification of Diseases (ICD 11)	1.6%
Quality	Guidance	17	M10 - M4Q(R2) EWG - Q1 EWG - Q12 - Q12 IWG - Q13 - Q2(R2) - Q14 - Q3C (R9) - Q3D (R2) - Q3E EWG - Q4A - Q4B(R1) - Q5A(R2) IWG - Q5A(R2) - Q6(R1) EWG - Q9(R1)	37.7%
Quality	Standards and Norms	1	Enhancing Regulatory Reliance and Agility	
Quality	Training	5	Q13 IWG - Q2(R2)/Q14 IWG - Q9(R1) IWG - PIC/S Webinar for Inspectors Training on ICH Q12 - Training activities relating to ICHQ9	

Guidance development is the primary outcome of nearly all ICH activities (85.2%), aligning with its core mission of establishing technical guidelines (3). A significant portion of ICH’s recent efforts are directed toward topics related to Quality.

#### 3.1.3 Pharmaceutical Inspection Cooperation Scheme

In 1970, “The Convention for the Mutual Recognition of Inspections in Respect of the Manufacture of Pharmaceutical Products” was created, which later became PIC (Pharmaceutical Inspection Convention) (10). The activities of PIC/S are (Table 3).

**Table 3:** PIC/S topic domains and outputs by project.

Topic domain	Output	Number of projects	Summary of project types	% of PIC/S activities by topic
Convergence and reliance	Collaborative work	2	Define PIC/S′ strategy and future policy and make proposals on how to improve the structure and the operation of PIC/S and discussing new projects	50.0%
Convergence and reliance	Guidance	2	PIC/S guidance on GMP Inspection Reliance based on ICMRA draft to maximize inspection resources for GMP compliance - Memorandum of Understanding (MoU) between PIC/S and ICH	
Convergence and reliance	Information	1	Monitor PIC/S′ public relations, exchange of information and define a communication strategy in order to better promote PIC/S and its key role in the field of inspections and Expert Circles (SCEC) - Facilitate discussions among Inspectors specialized in a specific area of GMP (Blood, Computerized Systems, API, Quality Risk Management…)	
Convergence and reliance	Training	2	Training Competent Authorities on GMPs and, in particular, training Inspectors and Training on GMP Compliance process to apply an inspection system to ensure the proper implementation of the Scheme	
Quality	Guidance	2	High and harmonized GMP standards and guidance documents for inspection practices - WHO good manufacturing practices for sterile pharmaceutical products	50.0%
Quality	Standards and Norm	1	Enhancing Regulatory Reliance and Agility (PQKMS)	
Quality	Training	3	Expert Circle on Quality Risk Management - PIC/S Webinar for Inspectors Training on ICH Q12 - Training activities relating to ICHQ9	
Quality	Collaborative Work	1	Working group on Distant Assessment	

PIC/S seeks to harmonize global GMP inspection standards by offering inspector training, developing common guidelines, and enhancing cooperation among regulatory authorities (10). This study confirms that PIC/S’s activities align with its mission, focusing on Convergence & Reliance and Quality. This is achieved primarily through Training (35.7%), Guidance (28.6%), collaborative work (21.4%), and establishing standards and norms and information (7.1% each), showing that PIC/S’s actions effectively reflect its goals in practice.

#### 3.1.4 International Pharmaceutical Regulators Programme

The International Pharmaceutical Regulators Programme (IPRP) is the result of the merger in 2018 of the International Pharmaceutical Regulators Forum (IPRF) and the International Generic Drug Regulators Programme (IGDRP) (11), the members are regulators only. 35 IPRP activities were identified and are displayed in the Table 4.

**Table 4:** IPRP topic domains and outputs by project.

Topic Domain	Output	Number of projects	Summary of project types	% of IPRP activities by topic
Convergence and reliance	Collaborative work	4	Collaboration for a Strategic Vision and Management Committee on Reliance - e-Labelling - Environmental Risk Assessment, Challenges in the implementation of ICH guidelines - Patient’s experience - Maintain knowledge of regulatory activities in participating regions, Collaborations and information sharing with other international and regional bodies, Identify topics for regulatory convergence or harmonization	17.1%
Convergence and reliance	Information	2	IPRP Questions and Answer document on Reliance - WHO questionnaire analysis	
Digital	Information	1	AI	5.7%
Digital	Collaborative work	1	e-Labelling	
Generics and biosimilars	Collaborative work	1	Reflection paper on extrapolation of indications in authorization of biosimilar products - Highlight NRAs harmonized scientific considerations on the extrapolation of indication(s) for biosimilar products - Increasing the Efficiency of Biosimilar Development Programs — Reevaluating the Need for Comparative Clinical Efficacy Studies	28.6%
Generics and biosimilars	Guidance	2	Alternative comparator product policies - BCS-based biowaivers	
Generics and biosimilars	Information	5	Acceptability of foreign comparator products in bioequivalence studies - Additional strength biowaivers - Bioequivalence study design - Biowaivers by dosage form - IPRP BWG regulatory information sharing platform	
Generics and biosimilars	Training	2	Primer on Biosimilar-Related regulatory topics for regulatory reviewers - Training manual for regulatory reviewers (biosimilar monoclonal antibodies)	
Innovative therapies	Collaborative work	1	Regulatory collaboration	28.6%
Innovative therapies	Guidance	4	Gene therapy working group & Cell therapy working group: Compilation of Guidance, Guideline and Reflection Paper - Initiate Reflection Paper (RP) on Long-Term Follow-up (LTFU) for patients receiving gene therapy products - Product Specific guidance subgroup - Raw Materials Reflection Paper	
Innovative therapies	Information	3	Exchange scientific and regulatory considerations for generic and follow-on nanomedicines - Information sharing and mapping: Liposomes - Regulatory Framework Project	
Innovative therapies	Standards and Norms	1	Lipid Nanoparticle Subgroup	
Innovative therapies	Training	1	Training on the lipid nanoparticle platform technologies used to deliver mRNA / DNA vaccines, genes therapies and siRNA	
Non-clinical	Information	1	Environmental Risk Assessment	2.9%
Pharmacovigilance	Information	1	Define regulatory tasks and terminology relevant to PV	2.9%
Quality	Guidance	2	The IPRP QWG Pilot Project of Active Substance Master File (ASMF)/Drug Master File (DMF) Database - Guidance for Quality assessors: Drug Product	14.3%
Quality	Standards and Norms	2	Ensure the awareness and understanding of the standards - Enhancing Regulatory Reliance and Agility	
Quality	Information	1	Survey on Administrative Procedures and Terminologies for Quality Variations/Post-Approval Changes	

IPRP aims to foster an environment for regulatory members and observers to share information on shared concerns, facilitate collaboration, and promote convergence of regulatory approaches for human pharmaceutical products (11). The study over the past 6 years shows that IPRP members have focused primarily on Generics and Biosimilars and Innovative Therapies, followed by Convergence & Reliance. Most outputs have been in the form of information (40.0%), guidance (22.9%), and collaborative work (20.0%), reflecting IPRP’s goal of information exchange on mutual interests among regulators. Unlike ICH, which focuses on technical guidelines, IPRP centers on sharing insights and approaches, with mature topics potentially transitioning to ICH if fully harmonized technical guidelines are warranted.

#### 3.1.5 International Conference of Medicines Regulatory Authorities

The International Conference of Medicines Regulatory Authorities (ICMRA) was established following discussions in May 2012 between 30 drug regulatory authorities (12). During the exchanges, the authorities highlighted the importance of promoting and coordinating joint work to strengthen the quality, safety, and efficacy of medicines globally, and more broadly the importance of harmonization to consider recognition procedures. This study of ICMRA activities identified the following:

ICMRA’s work is categorized into three main types of output: standards and norms (36.7%), collaborative work (33.3%), and guidance (20.0%). ICMRA focuses primarily on public health issues (60.0%), especially those related to COVID-19 during the pandemic, where it tried to define standards and norms among the different country members. Then ICMRA works on other topic areas such as clinical (13.3%), quality (10.0%), convergence and reliance, digital (6.7%) and pharmacovigilance (3.3%). It does not cover areas such as Generics & Biosimilars, innovative therapies, medical devices, or non-clinical subjects (Table 5).

**Table 5:** ICMRA topic domains and outputs by project.

Topic domain	Output	Number of projects	Summary of project types	% of ICMRA activities by topic
Clinical	Collaborative work	3	Evolution of clinical trials - Meetings and teleconferences - Statement on international collaboration to enable RWE for regulatory decision-making	13.3%
Clinical	Standards and Norms	1	Joint Statement on transparency and data integrity International Coalition of Medicines Regulatory Authorities (ICMRA) and WHO	
Convergence and reliance	Guidance	2	Statement on the value of regulatory reliance - PIC/S guidance on GMP Inspection Reliance based on ICMRA draft to maximize inspection resources for GMP compliance	6.7%
Digital	Collaborative work	1	Use of artificial intelligence (AI) and machine learning (ML) in the regulation of medicines	6.7%
Digital	Guidance	1	Working practices - Strategic priority on innovation (Perceived issues, 3D Bio-Printing, Artificial intelligence - AI)	
Pharmacovigilance	Collaborative work	1	Vaccines sub working group	3.3%
Public health	Collaborative work	4	Report from the ICMRA/WHO workshop on: Global perspectives on COVID-19 vaccines strain update Alignment on timing and data requirements - Statement on the outcomes of the ICMRA-WHO joint workshop on COVID-19 vaccines strain change - COVID-19 Vaccine Pharmacovigilance Network - ICMRA statement on COVID-19.	60.0%
Public health	Guidance	2	Framework for the Involvement of Health Authorities in the Management of Global Health Crises, Project based on public statement written in 2019, AMR project started in 2021	
Public health	Standards and Norms	9	COVID-19 vaccine trial designs in the context of authorized COVID-19 vaccines and expanding global access: ethical considerations - WHO-ICMRA joint statement on the need for improved global regulatory alignment on COVID-19 medicines and vaccines - List of COVID-19 master protocols - Query on acceptable clinical trial endpoints and outcomes for technical workshops on vaccines and treatments - ICMRA statement on clinical trials & ICMRA Statement on Need for Continued Focus on COVID-19 Therapeutics - Reflections on the regulatory experience of remote approaches to GCP and GMP regulatory oversight during the COVID-19 Pandemic - Report on the review of regulatory flexibilities/agilities as implemented by National Regulatory Authorities during Covid-19 pandemic - Deep dive report on the review of provisions and procedures for emergency authorization of medical products for COVID-19 among ICMRA members - Statement on continuation of vaccine trials	
Public health	Information	3	Statement for healthcare professionals: How COVID-19 vaccines are regulated for safety and effectiveness - Clinical trials inventory of actionable trials - ICMRA statement on the safety of COVID-19 vaccines	
Quality	Guidance	1	Facilitate the use of track and trace systems at global level	10.0%
Quality	Standards and Norms	1	Enhancing Regulatory Reliance and Agility (PQKMS)	
Quality	Collaborative work	1	Working group on distant assessment	

#### 3.1.6 International Medical Device Regulators Forum

The International Medical Device Regulators Forum was established in October 2011. Representatives of WHO and medical device regulators came together to form an organization with the aim of accelerating the harmonization of the medical device regulatory model (13). This goal is part of the context of the globalization of the production of medical devices, but also of the arrival on the market of new technologies related to health. IMDRF’s activities and actions are as follows:

IMDRF (Table 6) engages in activities related exclusively to medical devices (100%). The output is mainly in creating guidance (55.6%), Information (22.2%) and standards & norms (22.2%), facilitating a working group, and creating a regulatory framework.

**Table 6:** IMDRF topic domains and outputs by project.

Topic domain	Output	Number of projects	Summary of project types	% of ICMRA activities by topic
Medical device	Guidance	5	Quality Management Systems - Medical Device Cybersecurity Guide - Unique Device Identification (UDI) Application Guide - Personalized Medical Devices (PMD) - Good Regulatory Review Practices working group	100%
Medical device	Standards and Norms	2	Adverse Event Terminology - Artificial Intelligence working group	
Medical device	Information	2	Regulated Product Submission Working Group, Software as a medical device working group	

#### 3.1.7 Overall view by regulatory organization

**Figure 1:**
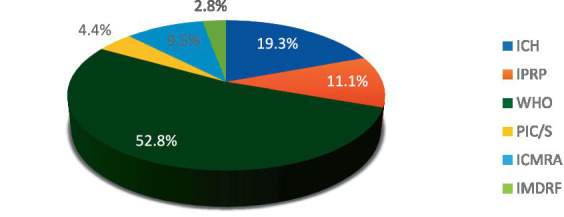
Distribution of overall projects by organization.

Figure 1 shows the distribution of all listed projects. Out of a total of 316^1^ projects, the majority are managed by the WHO.

In Figure 1, WHO represents most projects, with 52.8% of all activities followed by ICH with 19.3%. The remaining activities are distributed among the other organizations, with 11.1% of activities carried out by the IPRP, 9.5% by the ICMRA, and 4.4% by PIC/S, and 2.8% by IMDRF. It can be surmised that not all organizations engage in the same proportion of activities.

Having established that the proportion of associated activities differs between organizations and topics, we can now undertake a comparative analysis of the distribution of topics domain.

#### 3.1.8 Overall view of projects by topic domains

**Figure 2:**
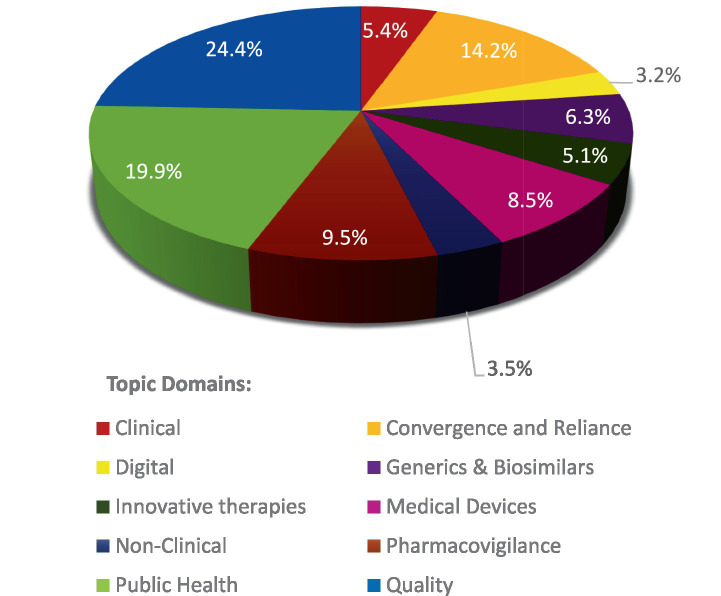
Distribution of projects by Topic Domains across the Organizations.

Figure 2 summarizes the topic domains across all the Organizations, the overall distribution is as followed:

Figure 2 shows that focus areas for many organizations are Quality (24.4%) and Public Health (19.9%). Convergence & Reliance (14.2%), Pharmacovigilance (9.5%), Medical Devices (8.5%), Generics and Biosimilars (6.3%), clinical (5.4%) and non-clinical (3.5%) are other topics of interest by International Organizations in descending order. However, there are emerging trends in Innovative Therapies (5.1%) and Digital (3.2%). This distribution of subjects is uneven, indicating that some areas receive more attention and projects than others, though we note this analysis captures only our primary grouping/categorization and that a number of projects touch on different Topic Domains in their scope.

**Figure 3:**
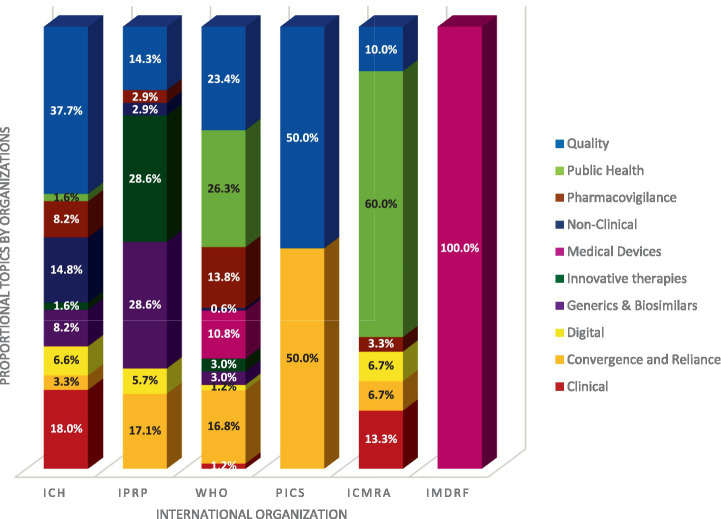
Distribution of topics domain by organizations.- ICH total of projects: 61 (23 in quality;1 in Public Health;5 in PV;9 in non-clinical;1 in innovative therapies;5 in Generics & Biosimilars;4 in digital;2 in convergence & reliance;11 in clinical), IPRP total of projects: 35 (5 in quality;1 in PV;1 in non-clinical;10 in innovative therapies;10 in Generics & Biosimilars; 2 in digital; 6 in convergence and reliance), WHO total of projects: 167 (39 in quality;44 in public health;23 in PV;1 in non-clinical;18 in medical devices;5 in innovative therapies;5 in Generics & Biosimilars;2 in digital;28 in convergence and reliance;2 in clinical), PIC/S total of projects: 14 (7 in quality;7 in convergence and reliance), ICMRA total of projects: 30 (3 in quality;18 in public health;1 in PV;2 in digital;2 in convergence and reliance;4 in clinical), IMDRF total of projects: 9 (9 in medical devices) – To realize this figure a normalization of the percentage has been done between organizations.

We examined how different organizations distribute their work according to subject matter. Figure 3 reveals the proportion of work each organization dedicates to various topics, allowing us to see which organizations are mainly engaged in specific areas.

Figure 3 highlights two key observations. First, some topics are only addressed by a minority of international organizations, such as innovative therapies and Generics and Biosimilars for IPRP, non-clinical topics for ICH, and medical devices for IMDRF. In contrast, some topics, such as quality and convergence and reliance are addressed by nearly all organizations. In areas of overlap there may be value in further examining the scope and types of projects to ensure there is no duplication of efforts and to enable potential efficiencies in activities.

The second observation is that PIC/S and IMDRF maintain a strong focus on their respective expertise: PIC/S on quality/GMP and convergence & reliance, and IMDRF on medical devices. In contrast, ICH, IPRP, WHO, and ICMRA engage with a broader spectrum of topics.

### 3.2 Output of regulatory organization

#### 3.2.1 Distribution of outputs

This study explored the nature of actions and outputs resulting from the work of various organizations, considering their unique contexts. Despite the varied distribution of subjects across organizational activities, an important question arises: to what extent do organizations working on the same subject produce similar types of work? The first step in this analysis was to examine the distribution of different types of activities produced by these organizations.

**Figure 4:**
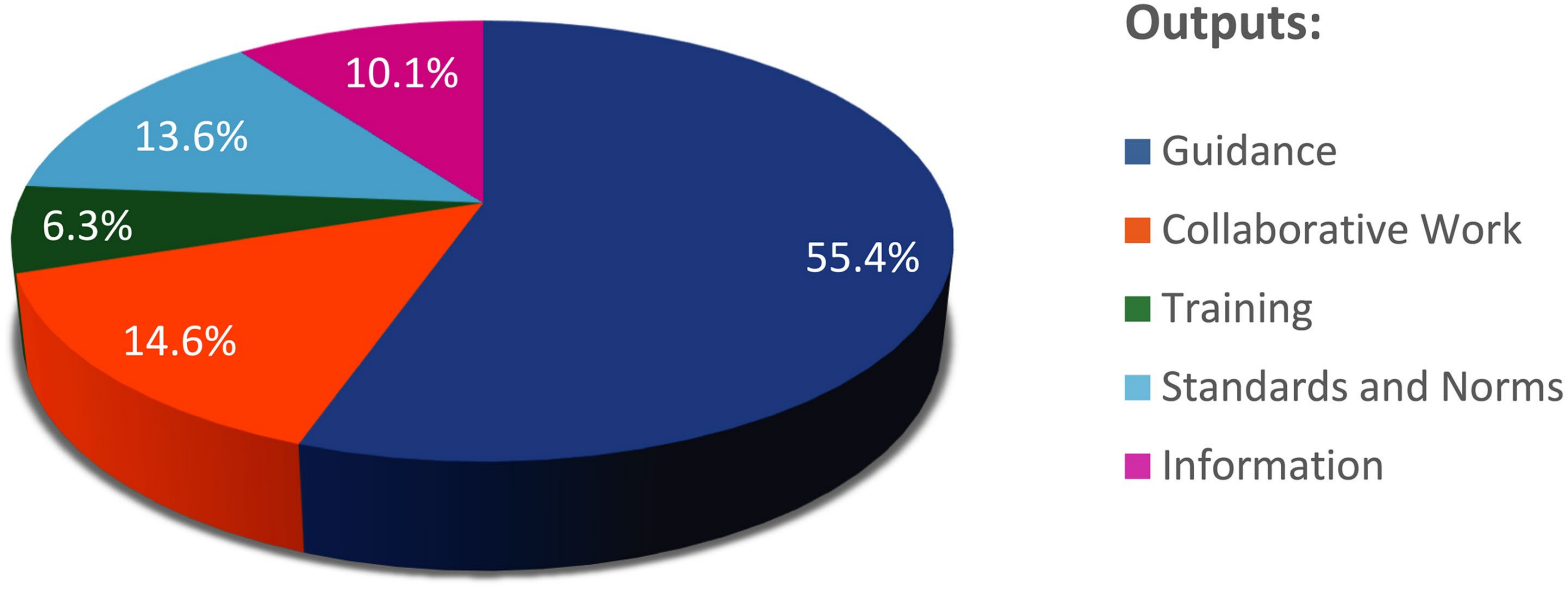
Distribution of projects by outputs across the Organizations.

As shown in Figure 4, most activities (55.4%) lead to the establishment of guidance, which is expected given the regulatory nature of these organizations. Organizations’ outputs relate to collaborative work in 14.6% of the activities within scope, Standards and Norms in 13.6%, Information in 10.1% and then, training in 6.3%.

#### 3.2.2 Distribution of outputs among various organizations

**Figure 5:**
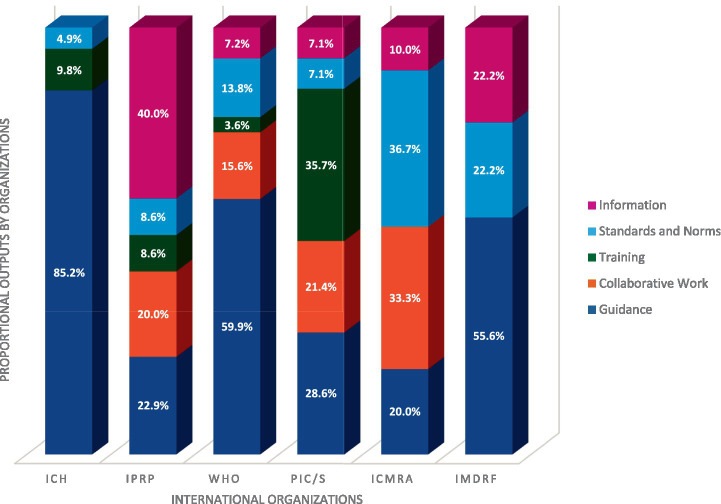
Distribution of outputs by Organization – ICH total of outputs: 61 (3 in standards and norms;6 in training;52 in guidance), IPRP total of outputs: 35 (14 in information; 3 in standards and norms; 3 in training; 7 in collaborative work; 8 in guidance), WHO total of outputs: 167 (12 in information;23 in standards and norms;6 in training; 26 in collaborative work; 100 in guidance), PIC/S total of outputs: 14 (1 in information;1 in standards and norms;5 in training;3 in collaborative work;4 in guidance), ICMRA total of outputs: 30 (3 in information;11 in standards and norms;10 in collaborative work;6 in guidance); IMDRF total of outputs: 9 (2 in information; 2 standards and norms; 5 in guidance) – To realize this figure a normalization of the percentage has been done between organizations.

Figure 5 shows that the distribution of these outputs varies from organization to organization. IPRP, PIC/S, and ICMRA produce a mix of all outputs while ICH, WHO and IMDRF are primarily producing guidance. Training remains the least represented output overall, despite the fact that it is more prominently developed by PIC/S [which is aligned with one of the main focuses of PIC/S: inspector training (10)].

Figure 5 also shows that all the organizations are involved in the development of guidance documents, with ICH having the highest proportion at 85.2%.

### 3.3 Link between organizations and topics

#### 3.3.1 Collaborations

The analysis of cross-collaboration provides valuable insights into how organizations leverage their collective strengths. A comprehensive review of collaborative activities revealed the extent and nature of inter-organizational partnerships. This examination not only quantified the number of cross-collaborations but also illuminated the primary domains where these synergies occur. Furthermore, it highlighted the preferred types of outputs resulting from these joint efforts, offering a clear picture of how organizations strategically combine their expertise to achieve common goals and drive innovation in specific fields. Of note, only 18 of the 316 projects (6%) represent cross collaboration between organizations. These collaborative projects address topics including quality, convergence & reliance, public health, and clinical aspects.

In the area of quality, WHO and PIC/S are collaborating on a project to develop guidance on Good Manufacturing Practices for sterile pharmaceutical products (14). PIC/S is also working with ICMRA through their working groups on distant assessments, where exchanges have led to a collaborative effort between these two working groups (15). Additionally, PIC/S collaborates with ICH on two training initiatives: a webinar for inspectors on ICH Q12 (16) and training activities related to ICH Q9 (17). Two other quality-related projects output to produce standards and norms. The first involves four organizations—ICMRA, ICH, PIC/S, and IPRP—working on a harmonization and convergence plan to advance the development of a regulatory Pharmaceutical Quality Knowledge Management (PQKM) capability (18). The second project, a joint initiative between IPRP and WHO, focuses on the Identification of Medicinal Product (IDMP) Working Group (19).

In the area of convergence and reliance, a Memorandum of Understanding (MoU) between PIC/S and ICH has been signed to facilitate collaboration on several ICH Guidelines relevant to inspectorate activities and to provide training for both assessors and inspectors (20). PIC/S is also participating in a collaborative effort to develop guidance on GMP inspection reliance, based on a draft by ICMRA, with the goal of optimizing inspection resources for GMP compliance at overseas facilities (15). Additionally, ICMRA and WHO collaborated to organize a workshop on global perspectives for updating COVID-19 vaccine strains, aimed at aligning timing and data requirements; this initiative fostered closer cooperation between the two organizations (21). WHO is also engaged in a joint activity with IPRP to share findings from the analysis of responses to the WHO questionnaire on reliance (22).

In the area of public health, WHO and ICMRA are collaborating on COVID-19 vaccine strain updates through workshops that have led to collaborative efforts among their member organizations (23, 24). Additionally, WHO and ICMRA are working together on four projects to establish standards and norms related to COVID-19: one focused on trial designs for COVID-19 vaccines in the context of existing authorizations and global access, considering ethical implications (25), a joint statement calling for improved global regulatory alignment on COVID-19 medicines and vaccines (26), a report on the review of regulatory flexibilities/agilities as implemented by National Regulatory Authorities during Covid-19 pandemic (27) and a deep dive report on the review of provisions and procedures for emergency authorization of medical products for COVID-19 among ICMRA members (28). WHO is also collaborating with ICH on establishing standards and norms through the development of the International Classification of Diseases (ICD-11) (29). Furthermore, WHO and ICMRA have jointly issued an informational statement aimed at healthcare professionals, explaining the regulatory measures for ensuring the safety and effectiveness of COVID-19 vaccines (30).

In the clinical domain, WHO and ICMRA have collaborated on transparency and data integrity through a joint statement, fostering cooperative efforts between the two organizations (31).

In areas of overlap, the scope and types of projects described above indicate that cooperation between these international regulatory organizations regularly occurs. Further targeted cooperation between these organizations, leveraging their core roles may accelerate delivery of shared topics and generate potential efficiencies for participating regulators.

#### 3.3.2 Deep dive on reliance

We also wished to examine if a high level of activity on one topic by a number of international organizations correlated with increased utilization of reliance pathways. An analysis was undertaken on one of the main topics shared by a large part of these international organizations: convergence and reliance in 10 countries.

**Figure 6:**
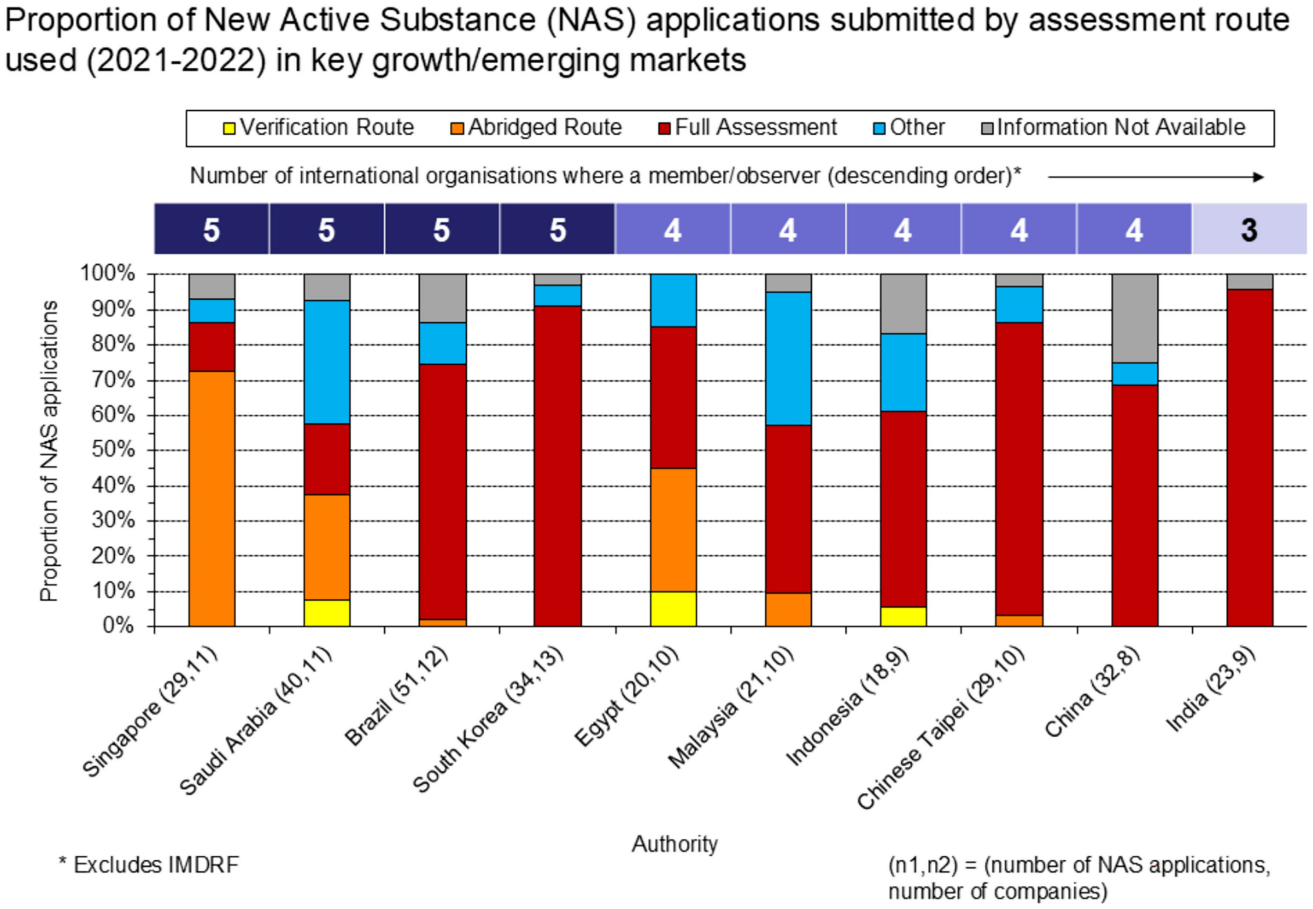
Proportion of NAS applications submitted, by assessment route used (2021–22).

Increased engagement in international organizations by the regulatory authorities in these countries appears to be correlated with higher proportions of applications submitted through reliance routes (Figure 6). This association may be facilitated by the alignment of jurisdictions with internationally recognized standards and technical guidelines, which helps build the trust necessary for reliance pathways to operate effectively. Jurisdictions that are involved in organizations like ICH and WHO often adopt these bodies’ guidelines and best practices, creating a foundation for harmonized or converged regulatory processes which can support reliance activities (1). We note however, there is a converse view and increased use of reliance and convergence may also facilitate or encourage participation of regulatory authorities in these international organizations.

The Certificate of Pharmaceutical Product (CPP) is a tool that can support reliance when used in conjunction with approval by a reference agency. For the jurisdictions our sample (Figure 6), CPPs were submitted for 63% of the NAS applications (CPP data not shown). Given that 59% of the NAS applications in our sample were identified as undergoing a full assessment, this suggests CPPs were frequently submitted for “full” reviews. For Egypt and Saudi Arabia specifically, CPPs were submitted for a high proportion of applications undergoing full assessments (88 and 63%, respectively). Notably, CPPs were submitted for a nominal proportion of applications to Singapore CPPs, consistent with the agency not requiring a CPP for regulatory submissions.

To explore if the date of joining an international organization showed any correlation with time to dossier submission, a trend analysis of submission lag was mapped against the commencing of involvement with ICH, as a member or observer, by the regulatory authority. Given ICH’s facilitation of technical harmonization and its potential to enable more efficient global development and regulatory review.

**Figure 7:**
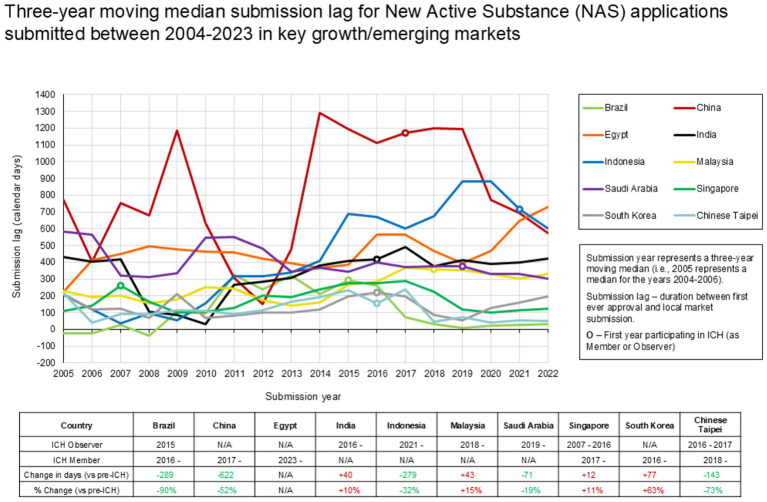
Three-year moving median submission lag of NAS applications submitted (2004–2023).

Figure 7 illustrates how submission lag (the time between first market approval and submission to the local market) has changed following a jurisdiction’s involvement with ICH, either as a member or observer. The data show a general trend of lower median submission lag following involvement with ICH, i.e., pre-ICH vs. 2022. Examples of this include China (−622 days), Brazil (−289 days), Indonesia (−279 days), Chinese Taipei (−73 days), and Saudi Arabia (−71 days), suggesting that alignment with international guidelines may streamline the submission process. Whilst submission lag has increased to some markets following involvement with ICH, it is noted that the % change in median submission lag (vs pre-ICH) is generally +15% or lower in those markets, except for South Korea (+63%).

### 3.4 Country representation in the various organizations

**Figure 8:**
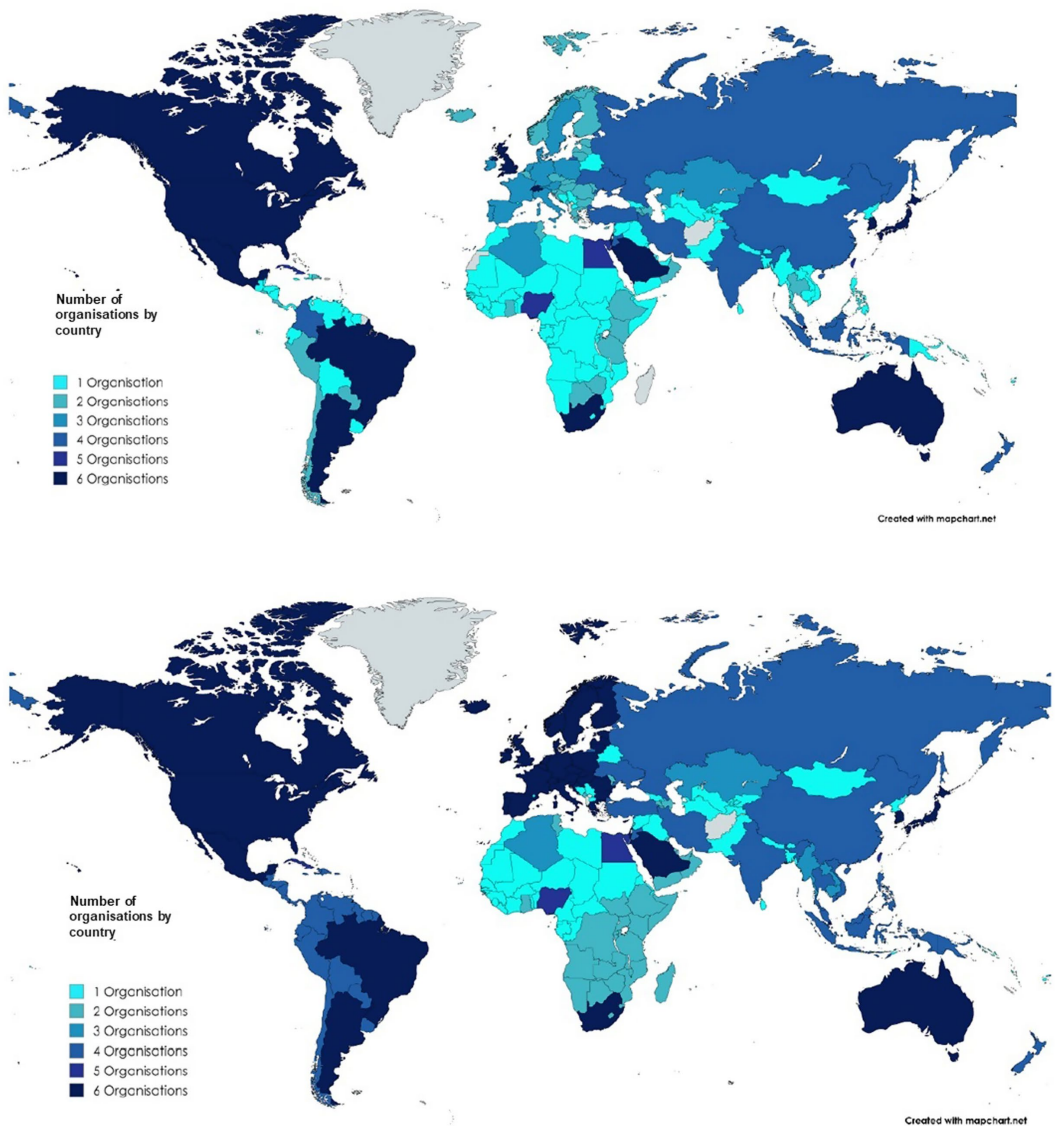
World map without regional harmonization initiative (up) and with Regional Harmonization Initiatives (down) - List of RHI: Association of Southeast Asian Nations (ASEAN), Global Harmonization Working Party (GHWP), African Medical Devices Forum (AMDF).

An analysis was conducted to understand the representation of countries in international organizations. The aim was to compare the number of countries that are members of international organizations and the number of Regional Harmonization Initiatives (RHI) that are also members of international organizations; and to see whether participation in RHIs increases the number of memberships in international organizations at the country level. Two maps (Figure 8) allow to see the impact of the number of adhesions to international organizations first at a countrywide level and then at an RHI level.

At a regional level, observations indicate that regional harmonization initiatives have a positive impact on the involvement of countries in international regulatory organizations in all regions, except North America where there is no impact since both countries US and Canada are already very active in international organizations.

The two world maps illustrate worldwide changes in membership numbers through regional harmonization initiatives. As demonstrated in previous paragraphs, the impact is visible in Africa, Europe, the Middle East, Latin America and the Western Pacific.

By analyzing these figures, ICH member countries appear to be involved in more organizations as a single country (thus without RHIs) compared to the other countries.

Table 7 highlight membership numbers and proportions by membership, comparing ICH member or observer countries with non-member countries. More than 50% of ICH member countries are members of more than half of the organizations, while the vast majority (68.1%) of non-ICH member countries are members of only one organization. It is also noted that out of 38 ICH member countries, more than a quarter (36.8%) are members of all organizations, while out of the 185 non-ICH member countries, none are members of all 5 other organizations, and only one is a member of 4 other organizations. Finally, ICH member countries are on average members of 3.42 other organizations, while non-ICH member countries are members of 1.37 other organizations, with this average gap being significant with a *p-value* of less than 0.05. In conclusion, ICH member countries are members of more international organizations than other countries in the world. This may be explained in at least two ways, participation in a number of international organizations supports a move by regulatory authorities to undertake technical harmonization activities through participation in ICH. Or ICH membership signals a commitment by regulatory authorities to strengthen their regulatory frameworks including the technical guidelines of innovative medicines in line with global ‘best practices’ in parallel with broader participation in regulatory strengthening activities via the other organizations. In all likelihood, participation in ICH is underpinned by multifaceted drivers, however our results suggest it could be used as a surrogate marker for increased participation by regulatory authorities in international organizations.

**Table 7:** Comparison of the number of memberships of ICH member countries vs. non-ICH members countries.

* p < 0.05	Number of memberships (in addition to ICH)	0	1	2	3	4	5	Total	Average
Members of the ICH	Number of countries	0	5	7	7	5	14	38	3.42*
	% of ICH member countries	0%	13.2%	18.4%	18.4%	13.2%	36.8%	100%	
Non-ICH members	Number of countries	0	126	50	8	1	0	185	1.37*
	% of ICH member countries	0%	68.1%	27.0%	4.3%	0.5%	0%	100%	

## 4 Discussion

An examination of the various activities undertaken by international regulatory organizations demonstrates their largely complementary nature. The analysis reveals that the most active topics are associated with quality, public health, convergence & reliance, and pharmacovigilance. These topics are highly aligned with industry priorities as managing quality and safety of products globally, and throughout the product lifecycle, is highly complex. Streamlining and/or alignment of global requirements are an ongoing industry ask. In addition, efforts on convergence & reliance are also high priorities for industry since they can ease both new registration processes for early access to patients and post-approval changes. Public health efforts to prepare for future pandemics is also welcomed by all stakeholders, leveraging the learnings from Covid. Finally, it is important to clearly distinguish between Quality (GMP) initiatives and other areas of regulatory harmonization. GMP efforts have been in place for a much longer time and are generally more established than those related to pharmacovigilance or Good Clinical Practice. This distinction is critical to avoid conflating different levels of regulatory maturity of these disparate activities.

The priorities and activities of these international regulatory organizations also lay the foundation for more specific harmonization efforts, particularly in technical guidelines and practices.

The adoption of ICH technical guidelines by regulatory authorities helps harmonize the technical requirements for product approvals; however, while this is true in principle, the actual impact often depends on how these guidelines are interpreted and implemented. Divergent interpretations can sometimes lead to the development of country-specific requirements, which may inadvertently reintroduce regulatory divergence. In addition, the implementation of good practices (GxP) can help to further ensure consistency in assessment procedures. Subsequently, this may enable similar levels of risk tolerance across agencies, which can help to foster mutual trust between jurisdictions, as they recognize that consistent standards are being upheld by other agencies.

Greater alignment with global standards, and consequently greater trust between regulatory authorities, is essential for the implementation and function of reliance pathways. Enabling one regulatory authority to leverage the work from another trusted counterpart streamlines the review process and reduces duplication of effort. The flow from regulatory convergence, adoption of ICH guidelines and GxP, to similar risk tolerance, and then to mutual trust, creates the conditions where reliance pathways can succeed. Jurisdictions more engaged with international organizations are therefore better positioned to implement and utilize reliance pathways, which may lead to greater proportions of applications being submitted via these routes.

For the jurisdictions in our sample (Figure 6), the frequency of CPP submission to support the full assessment of an NAS suggests a disconnect between the use of documentation that can support reliance and how reliance is applied in practice. WHO guidance recommends that the use of reliance tools, such as the CPP, should support an abbreviated review focused on local context, rather than duplicating the assessment conducted by the Reference Regulatory Authority (RRA) (1). However, our analysis suggests that submission of a CPP may often be a procedural requirement rather than a tool to streamline regulatory review. This may reflect cautious regulatory approaches or legacy practices where agencies continue to require CPPs without consistent utilization within formal reliance practices. The high CPP submission rates for full assessments in Egypt and Saudi Arabia specifically may support this interpretation. When leveraged suitably, there is evidence that flexibility in the timing of CPP submission is also important: submission of the CPP after submission of the MAA, but before its approval, appears to be associated with shorter submission lag and faster rollout times (32). In contrast to many of the countries within the sample, Singapore does not require a CPP for NAS submissions, reflecting a different approach to agencies that often do so regardless of whether reliance is being formally applied. Singapore also represents an interesting case, given that formal reliance practices were established prior to joining ICH in 2017 (33). Together, these observations highlight the importance of distinguishing between formal reliance frameworks and how reliance is applied in practice, as agencies seek to align with international standards and improve regulatory efficiency.

These reliance pathways and international alignments not only facilitate national regulatory processes but may also impact the timeline between first market approval and submission to local markets.

Several factors contribute to the duration between first market approval and submission to the local market (i.e., submission lag). Company strategy plays a role, as companies may prioritize submissions to certain markets based on commercial considerations, operational capacity, or local expertise. Agency requirements, or local requirements, are another important factor. The adoption of common technical standards by regulatory authorities, such as those from the ICH, can reduce the number, complexity, and specificity of local requirements, thus reducing barriers to submission.

The country within our sample where the largest decrease in submission lag was observed following ICH membership was China (Figure 7). The accession of the China Food and Drug Administration (now NMPA) to ICH in 2017, and the adoption of harmonised guidelines such as ICH E17 - together with reforms enabling the acceptance of overseas clinical trial data, shortening of IND review timelines, and facilitation of China’s participation in multi-regional clinical trials (MRCTs) - may have enabled companies submit NDAs to China closer to their filings in the US or EU (34).

However, we note while ICH membership may correlate with a reduction in submission lag, this observation may also be explained by a regulatory authority committing to regulatory strengthening activities, which includes both participation in international organizations (and subsequent alignment with international standards) and a focus on national regulatory procedures, e.g., reducing review timelines. The use of reliance pathways offers the potential to further reduce submission lag. As trust builds between regulatory agencies through the adoption of common technical standards and good practices (GxP), reliance on approvals from trusted reference authorities becomes more feasible, allowing for faster regulatory reviews. This may encourage companies to submit earlier in markets where reliance is an option, with the knowledge that faster review processes could be leveraged vs., following standard national approval timelines. It is, however, important to acknowledge that utilization of reliance pathways can sometimes increase the time to local market submission following first market approval. This may occur if a company is waiting for a specific reference agency’s approval before submitting their application via a reliance pathway, as the reference agency may not always be the first to approve the product, and assessment reports and other reliance tools may not be immediately available. However, among the benefits of using reliance recovered from a survey performed by EFPIA, it was shown a reduction of timelines to approval of 95% (on 40 answers) and a reduction of number of questions from the relying agency by 86% (on 36 answers). The survey also highlighted persistent hurdles that hinder the benefits of unilateral reliance pathway, such as variability in documentation requirements, redundancy in the requested documents and a lack of consistency across the documents requested by each national regulatory authority (NRA) when relying on EMA assessment (35).

The concept of trusted reference authorities in reliance aligns with efforts by international organizations to identify and recognize regulatory bodies that consistently apply high standards. The has recently moved to a model of identifying and assessing “*WHO-listed authorities*” (WLA), via a Global Benchmarking Tool. The idea being to use this list for the review and subsequent registration of drugs in collaboration with these authorities. These authorities are therefore recognized by WHO to apply strict quality, safety and efficacy standards in their procedures for registering medicines and vaccines and alignment with certain international standards, i.e., specific ICH guidelines are assessed (36). This recognition of high-standard regulatory bodies by international organizations has significant implications for both pharmaceutical companies and regulatory authorities, both in the potential timings of submissions and subsequent availability of medicines. But also, critically in the role regulatory agencies wish to play in relationship to one another. Adoption of international guidelines builds a shared risk tolerance between agencies, which is the result of convergence of requirements, adopting common technical standards and GxP, which may enable not only unilateral reliance between reference and reliant authorities but supports building towards mutual reliance and/or mutual recognition mechanisms in the longer term.

The study highlights also the importance of examining the role and structure of international organizations in shaping the global regulatory landscape. The activities of international regulatory organizations often fall into the same categories as those of industry, reflecting areas of shared focus by regulator and regulated, even in fora where the industry does not have a seat.

New topics have also emerged in recent years, i.e., digital and innovative therapies, highlighting the constant evolution of scientific knowledge and the need to update regularly existing global and national regulations while also creating new guidelines for emerging topics. As more countries participate in international organizations, we would hope this may lead to more rapid adoption of aligned global standards in new areas, e.g., digital technologies, artificial intelligence and/or new modalities and prevent regulatory divergence appearing which would require subsequent global alignment activities at a later date.

Participation in international organizations by regulatory authorities can benefit all stakeholders and it is widely recognized that international collaboration strengthens regulatory systems across numerous domains. With that in mind, we note that, from a geographical point of view, countries have an advantage in being members of Regional Harmonization Initiatives (where available) as it allows them to be represented in more international regulatory organizations, and therefore to participate in international pharmaceutical regulation development. For example, ICH member countries are also members of more international regulatory organizations than other countries in the world, allowing them to be more involved in international pharmaceutical regulation and therefore better able to influence and shape it. Our results indicate ICH membership can be seen as a marker of participation in international activities to strengthen regulatory frameworks, as well as a commitment to harmonize with global technical standards. However, it should be noted that regulatory topics and activities in the pharmaceutical world sometimes face geopolitical, economic, and cultural difficulties that do not allow all countries to participate or play the same role in discussions that make it possible to change the global pharmaceutical framework.

This study, while insightful, has two main limitations. Firstly, projects were grouped into 10 broad categories, with each assigned to a single primary domain, potentially oversimplifying multi-domain projects. Secondly, outputs were classified into five categories, with only one primary output attributed per project, possibly overlooking multiple outcomes. Despite these simplifications, the global trends presented are expected to remain consistent, even with more detailed analysis. The current approach provides a clear overview without compromising the overall findings.

Finally, reliance has existed in certain regions of the world, and in Europe with mutual recognition procedures for 20 years. However, the COVID-19 pandemic accelerated the development and/or use of multiple reliance procedures around the world, enabling countries with less regulation to work with more stringent countries. It also enabled the authorities in these countries to increase their knowledge of new technologies, such as cell therapies, gene therapies and messenger RNA vaccines. This topic of reliance is of fundamental importance because it represents a revolution in the establishment of a new pharmaceutical framework that is more open at regional and global level.

## 5 Conclusion

International regulatory organizations are essential to enabling collaboration between nations, to ensure the safety, efficacy, and quality of medicines and healthcare products worldwide. They facilitate international cooperation by harmonizing regulatory standards and promoting the exchange of information and best practices among both countries and between medical technologies which supports crossing the boundaries between medicines and medical devices (such as ICH, PIC/S, and IMDRF) or between quality domains like CMC and GMP. The regulatory activities examined in this paper demonstrate a real synergy in the actual landscape of activities of these international regulatory organizations. Furthermore, the establishment of guidance is the main output which aligns with the regulatory nature of these organizations. However, as regulatory agencies capacities to participate in international organizations activities are finite, opportunities should be explored to build cross-organizational project plans on shared topics to maximize work and outputs across these different organizations.

Data suggests there may be a positive correlation between participation in international regulatory organizations and utilization of reliance pathways (Figure 6). This participation improves their ability to contribute to and benefit from international pharmaceutical regulation. This engagement should be encouraged in all the areas highlighted in this paper, as it promotes global standardization. Harmonized standards facilitate agency reliance and streamline dossier assessments, ultimately expanding global medicine reviews and access. The result is a more efficient, cohesive international regulatory landscape.

Finally, international regulatory organizations are also keeping pace with emerging trends, demonstrating their ability to adapt to the evolving regulatory landscape. Products developed by the pharmaceutical industry are increasingly complex and require the evaluating regulatory authorities to make rapid progress on these new technologies and to find synergies among themselves to guarantee rapid access to medicines for their countries, leading to a convergence of practices. Ongoing collaborative engagement both between regulatory authorities and between regulators and industry via international organizations will be critical keep pace with scientific progress, to help the convergence of practices, and to enhance the quality and effectiveness of global regulatory policies and procedures. Greater cross-organizational coordination will be essential to ensure that regulatory frameworks for current and emerging technologies are seamless, fit for purpose, and relevant, thereby avoiding fragmented or ineffective requirements across sectors that stifles innovation with the ultimate goal of accelerating patient access.

## Data Availability

The datasets presented in this study can be found in online repositories. The names of the repository/repositories and accession number(s) can be found in the article/supplementary material.

## Footnotes

^1^The total number of projects across all international regulatory organizations is 296. But Cross-collaboration projects are counted twice (or more) to enable the following graphics. For example, a project involving both WHO and ICMRA will be counted once under WHO and once under ICMRA.
